# Face mask recommendations in schools did not impact COVID-19 incidence among 10–12-year-olds in Finland – joinpoint regression analysis

**DOI:** 10.1186/s12889-023-15624-9

**Published:** 2023-04-21

**Authors:** Aapo Juutinen, Emmi Sarvikivi, Päivi Laukkanen-Nevala, Otto Helve

**Affiliations:** Department of Health Security, Finnish Institute for Health and Welfare, Mannerheimintie 166, 00271 Helsinki, Finland

**Keywords:** Finland, COVID-19, SARS-CoV-2, Face masks, Surveillance, Children

## Abstract

**Background:**

In autumn 2021 in Finland, a recommendation to use face masks was implemented nationwide in schools for pupils ages 12 years and above. While national guidelines were in form of recommendations, cities implemented mandatory masking in schools. Some cities extended this mandate for younger pupils as well. Our aim was to compare COVID-19 incidence among 10–12-year-olds between cities with different recommendations on the use of face masks in schools.

**Methods:**

COVID-19 case numbers, defined as positive laboratory verified SARS-CoV-2 test results, were obtained from the National Infectious Disease Registry (NIDR) of the Finnish Institute for Health and Welfare. Helsinki, Turku and Tampere were selected for comparison since the baseline COVID-19 incidence in the cities had been similar in August and September 2021. Helsinki and Tampere implemented the national recommendation on face mask use at schools, while Turku extended this to include those 10 years old and above, starting from the beginning of semester in early August. Age groups of 7–9-year-olds, 10–12-year-olds and 30–49-year-olds were included in the statistical analysis and moving averages of 14-day incidences per 100 000 inhabitants were used as a dependent variable. Joinpoint regression was used to estimate average percent changes (APC) and average daily percent changes (ADPC) in the 14-day incidences. Differences in the ADPC values between the cities were compared in one-month periods. We also calculated cumulative incidences from the beginning of August to the end of November in the cities by age group.

**Results:**

In August, the ADPC was highest in Turku (3.9) and lowest in Tampere (2.0), while in September, the ADPC was highest in Turku (-0.3) and lowest in Helsinki (-3.2) among 10–12-year-olds. In October, the ADPC was highest in Helsinki (2.1) and lowest in Turku (-0.2) and in November, the ADPC was highest in Turku (4.1) and lowest in Tampere (-0.5) among 10–12-year-olds. We also calculated cumulative incidences from the beginning of August to the end of November in the cities by age groups of 7–9 years, 10–12 years, and 30–49 years. The cumulative incidence was highest in Turku in all age groups and lowest in Tampere.

**Conclusions:**

According to our analysis, no additional effect was gained from mandating face masks, based on comparisons between the cities and between the age groups of the unvaccinated children (10–12 years versus 7–9 years).

## Background

In autumn 2021, the number of new COVID-19 cases was high globally [1]. In Finland, the Delta variant had begun to spread in June, and by the end of July, Delta was the dominant variant across the country. While nationally guidelines were in the form of recommendations, cities implemented mandatory masking in schools. At that time, face mask use was mandatory in schools for children aged 12 years and over. In some cities, this mandate was extended to pupils aged 10 years and above.

Face masks can have a protective effect in respect to the transmission of COVID-19 [2]. However, this protective effect can be significantly reduced or completely ignored if the face mask is not used properly [3]. This can be the case especially with young children.

There are no randomized controlled trials of masking in children, and The World Health Organization (WHO) stated that a risk-based approach should be applied to the decision to mask children between ages 6 and 11 years [4]. Little is known about the effectiveness of use of face masks among children on protecting them from viral transmission. In a recent Spanish study, face mask mandates in schools did not result in significant differences in COVID-19-transmission in schools [5]. However, studies with opposite results exist [6]. Our aim was to compare COVID-19 incidence among 10–12-year-olds between cities with different mandates on masking in schools.

## Methods

COVID-19 case numbers were obtained from the National Infectious Disease Registry (NIDR) of the Finnish Institute for Health and Welfare, where clinical microbiology laboratories report all positive SARS-CoV-2 tests with unique identifiers in a timely manner, including information such as date of birth, gender, and place of residence [7]. The NIDR is linked to the population data registry, enabling calculation of incidences. Moving averages of 14-day incidences per 100 000 inhabitants were used as a dependent variable in the statistical analysis. Joinpoint regression was used to estimate average percent changes (APC) and average daily percent changes (ADPC) in the 14-day incidences. Differences in the ADPC values between the cities were compared in one-month periods. Reporting ADPCs instead of APCs has several advantages, and it is a suitable measure to compare trends over a specific time period [8]. All figures were created using RStudio (R version 3.6.3) and all statistical analyses performed using the open source Joinpoint software (Joinpoint Regression Program, National Cancer Institute, USA, Version 4.9.0.0) as described previously [9].

Helsinki (population 661 887), Turku (population 195 818) and Tampere (population 245 230) were selected for comparison since the baseline incidence in the cities had been similar in August and September 2021. Helsinki and Tampere implemented the national recommendation on face mask use as mandates at schools, while Turku extended this to include those 10 years old and above allowing us to study the effect of masking children as a natural experiment. Minor local variation existed in Turku during a three-week period starting mid-October. Since November 11, the mask mandate for children aged 10 years and above was again valid in entire city of Turku.

Statistical analyses were performed in age groups of 7–9 years, 10–12 years and 30–49 years, with 10–12-year-olds being the most relevant group due to the experimental design. 7–9-year-olds were included in the analysis as a control group, representing pupils attending same schools as 10–12-year-olds, but without a mask mandate, and 30–49-year-olds representing the parents of children.

During autumn 2021 in Finland, school children (seven years and above) were recommended to be tested for COVID-19 even with minor symptoms. This testing policy was widely applied in the entire country. Confounding factors may exist, such as different guidelines for attending classes when displaying symptoms of COVID-19. Vaccination coverage of COVID-19 was not controlled in the statistical analysis. However, this mainly concerns 30–49-year-olds, since the vaccinations for children under 12 years of age were not conducted in Finland during the study period.

## Results

**Fig. 1:**
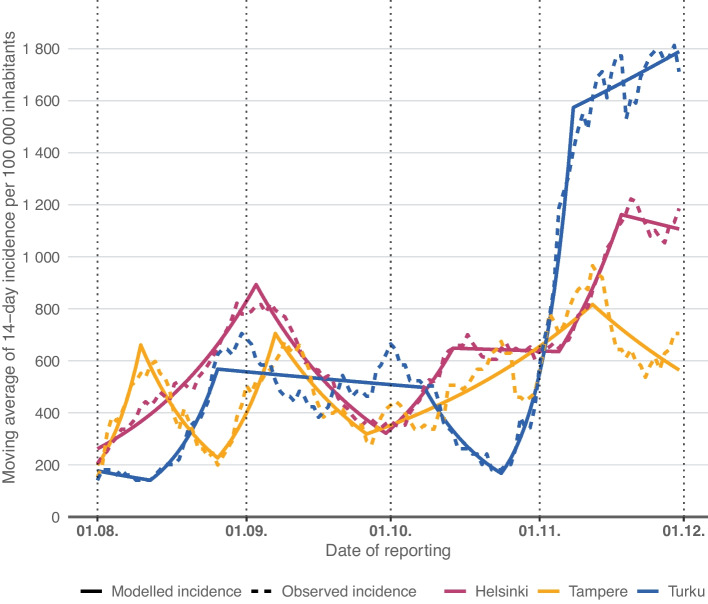
Moving averages of observed 14-day COVID-19 incidences per 100 000 inhabitants (dashed line) and modelled 14-day COVID-19 incidences per 100 000 inhabitants (solid line) based on estimated average percent changes (APC) in 10–12-year-olds in Helsinki and Tampere (face masks not mandated in schools in this age group) and in Turku (face masks mandated). Dotted vertical lines indicate the time periods in which the difference in the average daily percent changes (ADPC) were calculated

We compared the differences in trends of 14-day incidences per 100 000 inhabitants between Helsinki, Tampere and Turku among 10–12-year-olds, and among ages 7–9 and 30–49. Moving averages of observed 14-day incidences per 100 000 inhabitants and modelled 14-day incidences per 100 000 inhabitants based on estimated APCs among the 10–12-year-olds are presented in Fig. 1.

In August, the ADPC was highest in Turku and lowest in Tampere among the 10–12-year-olds (Table 1). There were no differences in the ADPCs between Helsinki and Turku. However, significant differences in the ADPCs were observed between Tampere and Helsinki, as well as between Tampere and Turku. Moving to September, Turku still held the highest ADPC, while Helsinki had the lowest. There were differences in the ADPCs between Helsinki and Turku and between Helsinki and Tampere, but not between Tampere and Turku. In October, the ADPC was highest in Helsinki and lowest in Turku. There were differences in the ADPCs between Helsinki and Turku and between Tampere and Turku but not between Helsinki and Tampere. Finally, in November, Turku once again had the highest ADPC, while Tampere had the lowest. There were differences in the ADPCs between each city.

**Table 1:** Estimated average daily percent changes (ADPC) and ADPC differences between the cities by age group during the autumn 2021

Age group	City	Estimate	August	September	October	November
7–9	Helsinki	ADPC	3.7 (*P* < 0.001)	-2.2 (*P* < 0.001)	0.4 (*P* = 0.04)	4.4 (*P* < 0.001)
	Tampere	ADPC	4.4 (*P* = 0.006)	-0.1 (*P* = 0.8)	1.6 (*P* < 0.001)	-1.1 (*P* < 0.2)
	Turku	ADPC	5.4 (*P* < 0.001)	0.2 (*P* = 0.4)	-1.0 (*P* = 0.004)	5.3 (*P* < 0.001)
	Helsinki vs. Turku	ADPC Difference	-1.7 (*P* < 0.001)	-2.4 (*P* < 0.001)	1.5 (*P* < 0.001)	-1.0 (*P* < 0.001)
	Helsinki vs. Tampere	ADPC Difference	-0.7 (*P* = 0.2)	-2.1 (*P* < 0.001)	-1.1 (*P* < 0.001)	5.5 (*P* < 0.001)
	Tampere vs. Turku	ADPC Difference	-1.0 (*P* = 0.1)	-0.3 (*P* = 0.6)	2.6 (*P* < 0.001)	-6.4 (*P* < 0.001)
10–12	Helsinki	ADPC	3.8 (*P* < 0.001)	-3.2 (*P* < 0.001)	2.1 (*P* < 0.001)	1.9 (*P* < 0.001)
	Tampere	ADPC	2.0 (*P* = 0.006)	-0.5 (*P* = 0.3)	2.0 (*P* < 0.001)	-0.5 (*P* < 0.2)
	Turku	ADPC	3.9 (*P* < 0.001)	-0.3 (*P* = 0.04)	-0.2 (*P* = 0.6)	4.1 (*P* < 0.001)
	Helsinki vs. Turku	ADPC Difference	-0.1 (*P* = 0.8)	-2.9 (*P* < 0.001)	2.3 (*P* < 0.001)	-2.2 (*P* < 0.001)
	Helsinki vs. Tampere	ADPC Difference	1.8 (*P* < 0.001)	-2.7 (*P* < 0.001)	0.1 (*P* = 0.7)	2.4 (*P* < 0.001)
	Tampere vs. Turku	ADPC Difference	-1.9 (*P* < 0.001)	-0.2 (*P* = 0.7)	2.2 (*P* < 0.001)	-4.6 (*P* < 0.001)
30–49	Helsinki	ADPC	-0.7 (*P* < 0.001)	-1.5 (*P* < 0.001)	1.2 (*P* < 0.001)	1.4 (*P* < 0.001)
	Tampere	ADPC	-3.0 (*P* < 0.001)	0.1 (*P* = 0.8)	0.9 (*P* < 0.001)	1.9 (*P* < 0.001)
	Turku	ADPC	0.8 (*P* < 0.001)	-0.6 (*P* = 0.003)	-1.3 (*P* < 0.001)	3.1 (*P* < 0.001)
	Helsinki vs. Turku	ADPC Difference	-1.5 (*P* < 0.001)	-0.8 (*P* < 0.001)	2.5 (*P* < 0.001)	-1.7 (*P* < 0.001)
	Helsinki vs. Tampere	ADPC Difference	2.3 (*P* < 0.001)	-1.6 (*P* < 0.001)	0.3 (*P* = 0.1)	-0.5 (*P* < 0.001)
	Tampere vs. Turku	ADPC Difference	-3.8 (*P* < 0.001)	0.7 (*P* = 0.1)	2.2 (*P* < 0.001)	-1.1 (*P* < 0.001)

Clegg et al. [8] have introduced details concerning the estimation and comparison

**Fig. 2:**
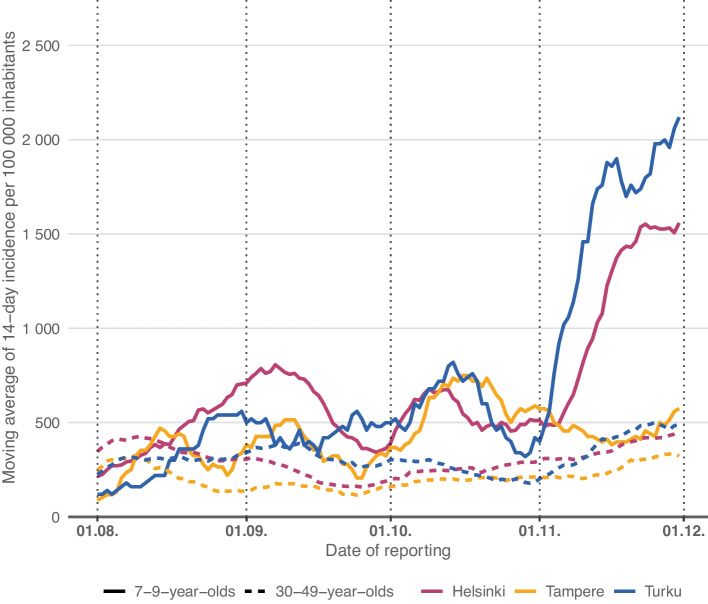
Moving averages of observed 14-day COVID-19 incidences per 100 000 inhabitants in 7–9-year-olds (solid line) and in 30–49-year-olds (dashed line) in Helsinki, Tampere and Turku

We also calculated and compared ADPCs between the cities among 7–9-year-olds and 30–49-year-olds, and the ADCPs and the differences between the cities were similar in all three age groups. Also, the incidence curve for 7–9-year-olds was similar to that of 10–12-year-olds. Yet, in the incidence curve of 30–49-year-olds, no such steep changes in November were observed in any of the cities (Fig. 2).

We also calculated cumulative incidences from the beginning of August to the end of November in the cities by age group (Table 2). The cumulative incidence was highest in Turku in all age groups and lowest in Tampere. Vaccination coverage of COVID-19 was not controlled in the statistical analysis. However, based on the data in the Finnish National Vaccination Register, we did not find relevant differences in the vaccination coverages among the 30–49-year-olds between the cities.

**Table 2:** Cumulative incidences and 95% CI per 100 000 inhabitants from August to November by city and age group

City	7–9-year-olds	10–12-year-olds	30–49-year-olds
Helsinki	6 500 (6 100, 6 800)	6 100 (5 800, 6 500)	2 600 (2 600, 2 700)
Tampere	4 000 (3 600, 4 500)	4 700 (4 200, 5 300)	1 800 (1 700, 1 900)
Turku	7 200 (6 400, 7 900)	6 500 (5 800, 7 200)	2 800 (2 700, 3 000)

## Discussion

In autumn 2021 in Finland, face mask use was recommended in schools for pupils ages 12 years and above. These recommendations were implemented as mandates in schools. In some cities, masking was mandated for younger pupils as well, allowing us to assess the impact of face mask use in schools for younger pupils as a supplementary pandemic control measure.

According to our analysis, no additional effect was gained from this, based on comparisons between the cities and between the age groups of the unvaccinated children (10–12 years versus 7–9 years). The ADPC among the 10–12-year-olds was highest in Turku almost every month except in October, when the ADPC was lowest in Turku. Also, the cumulative incidence was highest in Turku in all age groups.

According to a Spanish study mask mandates in schools were not associated with lower SARS-CoV-2 incidence or transmission, suggesting that this intervention was not effective [5]. Risk of transmission for children attending school increased with age. Transmission risk at schools, on the other hand, is lower than in households [10]. Our findings also support hypotheses that school infections broadly reflect community infections [11].

There have been conflicting results from studies examining the impact of mask mandates on COVID-19 cases. For example, a study conducted in the United States and published in late 2022, found that lifting mask requirements was associated with an increase in COVID-19 cases among students and staff [12]. However, this study aggregated data from all age groups, making it difficult to determine how the effects of mask recommendations might vary by age. In contrast, our study provides a more targeted analysis about the impact of mask mandates in primary schools. Additionally, the variant dominating was different between the studies, which could have influenced the findings.

The major limitation of our study is that schools are not the only place for children to have social contacts and to be exposed to SARS-CoV-2, and our study design did not allow tracking the places of transmission. However, the lower incidence in vaccinated adults would indicate a lower risk of infection at home. Therefore, one would expect to see some differences in the age-specific incidences if masking was an effective way to control transmission in schools. Asthe timing for these observations was during a high circulation of the Delta variant across the country,these results may not be valid during the Omicron era.

Moreover, we could not completely account for all confounding factors, like ascertainment of how strictly the face mask recommendations were followed in the schools. However, among these three regions, there was consensus on avoiding restrictions that had an impact on children’s everyday life, including free-time activities, and the implementation of infection control measures concerning hobbies was likely very similar between the cities. Guidelines for attending classes when displaying symptoms of COVID-19 were generally the same everywhere. Lastly, since this study is an observational study, causation cannot be inferred.

## Conclusions

Face mask recommendations in schools did not reduce COVID-19 incidence among 10–12-year-olds in Finland. This may indicate that COVID-19 cases in schools merely reflect community infections than school outbreaks. Future research in this area would benefit from prospectively controlled study design while examining impact of face mask use. Also, similar studies including older children would be valuable. Further research is needed to gain better understanding about the effectiveness of use of face masks among children.

## Data Availability

The data that support the findings of this study are not publicly available. The data can be availed from the corresponding author on reasonable request.

## Abbreviations

COVID-19: Coronavirus disease 19
APC: Average percent change
ADPC: Average daily percent change
